# Hemodynamic Effects of a Soluble Guanylate Cyclase Stimulator, Riociguat, and an Activator, Cinaciguat, During NO-Modulation in Healthy Pigs

**DOI:** 10.1177/1074248420940897

**Published:** 2020-07-14

**Authors:** Torvind Næsheim, Ole-Jakob How, Truls Myrmel

**Affiliations:** 1 Department of Clinical Medicine, Cardiovascular Research Groups, Faculty of Health Sciences, UiT The Arctic University of Norway, Tromsø, Norway; 2 Department of Anaesthesiology, University Hospital of North Norway, Tromsø, Norway; 3 Department of Medical Biology, Cardiovascular Research Groups, Faculty of Health Sciences, UiT The Arctic University of Norway, Tromsø, Norway; 4 Department of Cardiothoracic and Vascular Surgery, Heart and Lung Clinic, University Hospital of North Norway, Tromsø, Norway

**Keywords:** nitric oxide signaling, sGC-activator, sGC-stimulator, pulmonary hypertension, vasodilator

## Abstract

Cardiovascular diseases are often characterized by dysfunctional endothelium. To compensate for the related lack of nitric oxide (NO), a class of soluble guanylate cyclase (sGC) stimulators and activators have been developed with the purpose of acting downstream of NO in the NO-sGC-cGMP cascade. These drugs have been discovered using photoaffinity labeling of sGC and high-throughput screening of a vast number of chemical compounds. Therefore, an understanding of the integrated physiological effects of these drugs in vivo is necessary on the path to clinical application. We have characterized the integrated hemodynamic impact of the sGC stimulator riociguat and the activator cinaciguat in different NO-states in healthy juvenile pigs (n = 30). We assessed the vascular effects in both systemic and pulmonary circulation, the contractile effects in the right and left ventricles, and the effects on diastolic cardiac functions. Nitric oxide-tone in these pigs were set by using the NO-blocker l-NAME and by infusion of nitroglycerine. The studies show a more pronounced vasodilatory effect in the systemic than pulmonary circulation for both drugs. Riociguat acts integrated with NO in an additive manner, while cinaciguat, in principle, completely blocks the endogenous NO effect on vascular control. Neither compound demonstrated pronounced cardiac effects but had unloading effect on both systolic and diastolic function. Thus, riociguat can potentially act in various disease states as a mean to increase NO-tone if systemic vasodilation can be balanced. Cinaciguat is a complicated drug to apply clinically due to its almost complete lack of integration in the NO-tone and balance.

## Introduction

Cardiovascular diseases are often characterized by reduced production and sensitivity to nitric oxide (NO) in vascular tissue, and dynamic production and effect of this compound is a marker of a healthy vasculature. Nitric oxide is produced from an NO-synthase action on the amino acid arginine and acts downstream by stimulating soluble guanylate cyclase (sGC) in various cells, in particular, vascular smooth muscle cells.^1^ Guanylate cyclase, in turn, catalyzes the conversion of GTP to cGMP, an intracellular messenger acting through protein kinase G (PKG) and thus elicits a multitude of physiological responses including smooth muscle relaxation. The sensitivity of sGC to NO depends on the red-ox-state of sGC. In the physiologically reduced state, the NO-sensitivity is normal. In the oxidized heme-free state, NO cannot stimulate sGC.^2^

To circumvent the need for physiological NO production and a normal NO-affinity in the reduced sGC, a group of substances acting directly as pharmacological stimulators^3^ or activators^4^ of soluble guanylate cyclase has been developed. One of these compounds, the NO-stimulator riociguat (Adempas), is currently applied clinically in pulmonary hypertension.^5^ Although hemodynamically reasonable, the use of these compounds in clinical trials of heart failure has been hampered by extensive systemic hypotensive effects of both stimulators^6^ and particularly activators.^7-9^ However, the recently published VICTORIA study found a clinical benefit for the sGC stimulator vericiguat in patients with heart failure with reduced ejection fraction already on established guideline heart failure therapy.^10^

In theory, the sGC stimulators act directly on sGC and in concert with NO. On the other hand, sGC activators exert its activity independent of NO and has been found to bind primarily to “de-hemed” sGC in its oxidized forms. As stated, oxidized sGC is found predominantly in pathological tissues.^2,5,11,12^

There are many unknowns of the physiological effects and pharmacological applications of these compounds. From their biochemical profile, the activators should have minimal effects in normal vasculature. However, this has not been extensively addressed in intact, healthy animals. Furthermore, discrimination of relative effects in the pulmonary and systemic circulation have received little attention and will be valuable knowledge for their pharmacological application in various clinical settings. Finally, the extent to which the 2 different principal compounds interact with the NO-tone in vivo needs further clarification.

In this study, we have compared the circulatory effects of the sGC-stimulator riociguat, BAY 63-2521 (Bayer AG) and the activator cinaciguat, BAY 58-2667 (Bayer AG) in healthy, juvenile pigs. The study aimed to explore the relative systemic and pulmonary vascular effects of these 2 pharmacological compounds. Both drugs were applied in animals with normal, NO blocked, or NO-stimulated endothelial function. We also assessed whether the activator cinaciguat would, in fact, have effects in these healthy young animals with the presumed intact reduced form of sGC. Finally, the study aimed specifically to clarify how both drugs act on cardiac function.

## Material and Methods

### Experimental Animals

Thirty castrated male domestic pigs weighing 30 ± 5 kg were adapted to the Animal Department for 5 to 7 days. They were fasted overnight before experiments with free access to water. The experimental protocol was approved by the local steering committee of the National Animal Research Authority located at the Faculty of Health Sciences, UIT, The Arctic University of Norway. The FDF reference is 2012/55972.

### Surgical Preparation and Instrumentation

The pigs were premedicated with an intramuscular injection of 20 mg·kg^−1^ ketamine (Pfizer AS) and 1 mg of atropine (Nycomed Pharma). Anesthesia was induced by intravenous injection of 10 mg·kg^−1^ pentobarbital sodium (Abbott) and 0.01 mg·kg^−1^ fentanyl (Hameln Pharmaceuticals). The animals were normo-ventilated after tracheostomy. Fraction of inspired oxygen (FiO_2_) was chosen to maintain partial pressure of oxygen in the blood of 100 ± 2 mm Hg. FiO_2_ ranged from 0.20 to 0.30. Normo-ventilation was defined as an arterial PaCO_2_ of 40 ± 2 mm Hg. A central venous catheter was placed through the left internal jugular vein. Anesthesia was maintained throughout the experiment by a normative continuous infusion of 4 mg·kg^−1^ h^−1^ pentobarbital sodium, 0.02 mg·kg^−1^ h^−1^ fentanyl, and 0.3 mg·kg^−1^ h^−1^ midazolam (B. Braun). Anesthesia was titrated to avoid stress reactions during interventions. The circulating volume was maintained by a 10 to 20 mL·kg^−1^ h^−1^ continuous infusion of 0.9% NaCl supplemented with 1.25 g·L^−1^ glucose.

The animals received 2500 IU of heparin and 5 mg·kg^−1^ amiodarone (Sanofi-Synthelabo) to avoid blood clotting of catheters and to prevent cardiac arrhythmias. Hexamethonium chloride, trimethyl-[6-(trimethylazaniumyl)hexyl]azanium (Sigma-Aldrich) 15 mg·kg^−1^ was administered to avoid autonomous reflexes and single out vascular effects during interventions and measurements.^13^

A 7F manometer pressure-volume catheter (Millar MPVS Ultra) was inserted through an introducer sheath via the carotid artery into the left ventricle as proposed by Baan et al.^14^ The correct position was verified using 2-dimensional (2D) echocardiography and analysis of each volume segment of the catheter. The volume of the conductance catheter was calibrated at each point of the experiment by 2D echocardiography and stroke volume estimation by thermodilution. A 5F Swan-Ganz catheter (Edwards Lifescience Corp.) was advanced into the normal position in a pulmonary artery. A second balloon catheter was floated from the superior caval vein into the right ventricle for pressure measurements. Central venous pressure was measured in the right atrium. The systemic arterial pressure was assessed from a vascular catheter in the abdominal aorta. An 8F 50 mL IABP-balloon catheter (Maquet Cardiovascular) was introduced into the inferior caval vein and positioned just below the right atrium for intermittent preload reduction.

### Experimental Drugs

Riociguat and cinaciguat were obtained from Chemoki Synthesi-Tech as a dry powder. The drugs were solubilized as described in the study by Becker et al^15^: pH neutral solutions were prepared with dimethyl sulfoxide ([DMSO] Sigma-Aldrich) and a 1:1 solution of Transcutol, diethylene glycol ethyl ether (Sigma-Aldrich), and Cremophor, macrogolglycerol ricinoleate (Sigma-Aldrich). We used 5% Transcutol and 5% Cremophor solutions, and the ratio between DMSO, Transcutol, and Cremophor was 0.05:2.5:2.5. This solution was then further diluted with 0.9 mg·mL^−1^ NaCl to a final concentration of test drug of 0.01 to 0.1 mg·mL^−1^ of riociguat or cinaciguat, depending on the dose to be given. The maximum DMSO concentration was 0.02%. l-NAME, N(G)-nitro-l-arginine methyl ester (Sigma-Aldrich), was used as an NO synthase inhibitor^16^ and nitroglycerine ([NG] Takeda AS) as NO-donor.

### Experimental Protocol

Four and 3 pigs were used in a dose-response study for riociguat and cinaciguat, respectively (Table 1).

**Table 1.** Dose-Response Data for Riociguat and Cinaciguat.^a^

Riociguat, n = 4							
Dose in µg·kg^−1^	Before vehicle	Baseline	10	20	50	100	
MAP, mm Hg	89 ± 22	88 ± 21	87 ± 22	66 ± 11^b^	56 ± 13^b^	50 ± 9^b^	
MPAP, mm Hg	24 ± 3	25 ± 3	22 ± 2	23 ± 5	25 ± 7	25 ± 8	
SVR, dynes s^−1^ cm^−5^	1228 ± 171	1066 ± 248	957 ± 204	760 ± 180^b^	568 ± 124^b^	497 ± 161^b^	
PVR, dynes s**^−1^** cm**^−^** ^5^	205 ± 100	166 ± 109	146 ± 87	162 ± 76	179 ± 76	167 ± 65	
Cinaciguat, n = 3							
Dose in µg·kg^−1^·min^−1^	Before vehicle	Baseline	0.01	0.05	0.1	0.50	1.00
MAP, mm Hg	99 ± 20	96 ± 21	94 ± 22,	93 ± 18	84 ± 14	74 ± 12^b^	63 ± 6^b^
MPAP, mm Hg	20 ± 2	20 ± 2	20 ± 3	20 ± 3	20 ± 4	18 ± 2	18 ± 2
SVR, dynes s^−1^ cm**^−^** ^5^	1668 ± 164	1630 ± 199	1675 ± 347	1726 ± 287	1396 ± 152	1179 ± 251^b^	925 ± 232^b^
PVR, dynes s^−1^ cm^−5^	146 ± 34	148 ± 38	184 ± 72	170 ± 62	176 ± 116	130 ± 43	134 ± 8

Abbreviations: MAP, mean systemic arterial pressure; MPAP, mean pulmonary arterial pressure; PVR, pulmonary vascular resistance; SVR, systemic vascular resistance.

^a^ Values are mean ± SD. Mixed models statistics with pig identity as random effect was used. Significance levels are given between doses of test drug and before vehicle and between doses of test drug and baseline.

^b^ *P* < .05.

The following experiments were conducted using a repeated measurements design. After instrumentation, the pigs were allowed to rest for 30 minutes before baseline measurements.

**Figure 1.**
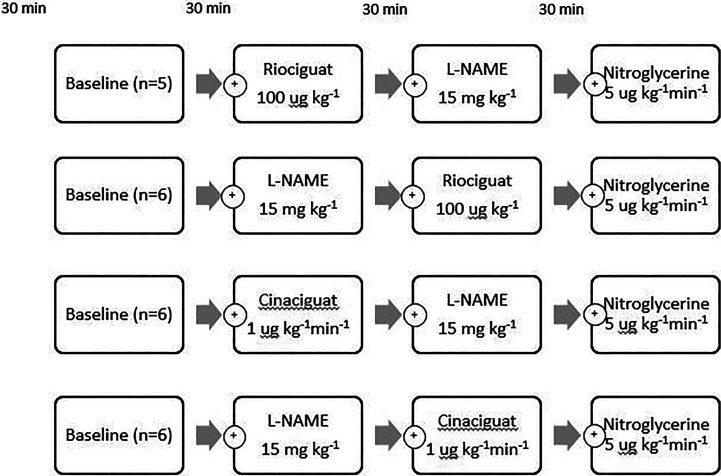
Outline of the 4 experimental groups. Following instrumentation and baseline recordings, NO modulation was induced by alternating administration of cinaciguat (Cina) 1 µg kg^−1^ min^−1^ or riociguat (Rio) 100 µg kg^−1^ combined with l-NAME 15 mg kg^−1^. At the end of all protocols remaining inducible NO response was assessed by NG 5 µg kg^−1^ min^−1^ infusion. l-NAME indicates N(G)-nitro-l-arginine methyl ester; NG, nitroglycerine; NO, nitric oxide.

Cinaciguat 1 µg·kg^−1^ min^−1^ was given as a continuous infusion and riociguat 100 µg as a bolus, l-NAME 15 mg·kg^−1^, and NG 5 µg·kg^−1^ min^−1^ was then given in alternating sequences in 4 different groups, each with 5 to 6 pigs (Figure 1). Administration form and timing of measurements were based on human pharmacokinetic studies.^17,18^ Equilibration after 30 minutes was observed between each measurement. The vehicle was given before baseline. Hemodynamic measurements were sampled at steady state with the ventilator in expiratory pause. Following hemodynamic measurements, the IABP-balloon in the inferior vena cava was inflated during data sampling to acquire pressure-volume data at different states of left ventricular (LV) work.^19^

### Registration of Data and Analysis

Data were sampled, digitized, and analyzed using ADI LabChart Pro software v 8.1.8. Cardiac output (CO) was obtained from thermodilution with the hardware Vigilance (Medtronic). Transthoracic echocardiography (Philips iE33) was done at all time points to measure end-diastolic and end-systolic diameters. End-diastolic volume (Ved) was calculated by Teicholts formula from echocardiographic end-diastolic diameter. End-systolic volume was calculated as the difference between end-diastolic volume and the stroke volume derived from the Swan-Ganz catheter. End-diastolic and systolic volumes were used to calibrate the conductance catheter at each time point. The time constant of relaxation was calculated with Weiss’ method from the exponential curve fitting of the LV pressure curve after dP/dt min (most negative pressure development during isovolumetric relaxation of the left ventricle). The Tau end point was set to 3 mm Hg above left ventricular end-diastolic pressure (LVEDP). Left ventricular stroke work (LVSW) and right ventricular stroke work (RVSW) were calculated as the respective areas of the pressure–volume relationship of the left and right ventricles, and preload recruitable stroke work (PRSW) were calculated from data derived from deloading of the heart as described by Burkhoff et al.^19^

### Statistical Analysis

All data in the main protocol have been tested to have normal or normality like distribution. The data are expressed as mean and SD in tables and figures. A linear mixed model^20^ with pig identity as subject (random effect, including intercept) and drug or drugs combinations as fixed effects was used to compare physiological values. The best covariance structure was found to be autoregressive, and a comparison of means was made by least significant difference. Restricted maximum likelihood ratio was used for model fitting. *P* values <.05 were regarded as statistically significant. All statistical analyses were conducted in SPSS 25.0.

## Results

Based on the dose–response studies, we chose the tested dose of riociguat and cinaciguat at 100 µg·kg^−1^ and 1 µg·kg^−1^·min^−1^, respectively. At these doses, the pigs showed a marked, but tolerable systemic vasodilatation, and remained hemodynamic stable with mean systemic arterial pressure (MAP) above 50 mm·Hg (Table 1).

**Figure 2.**
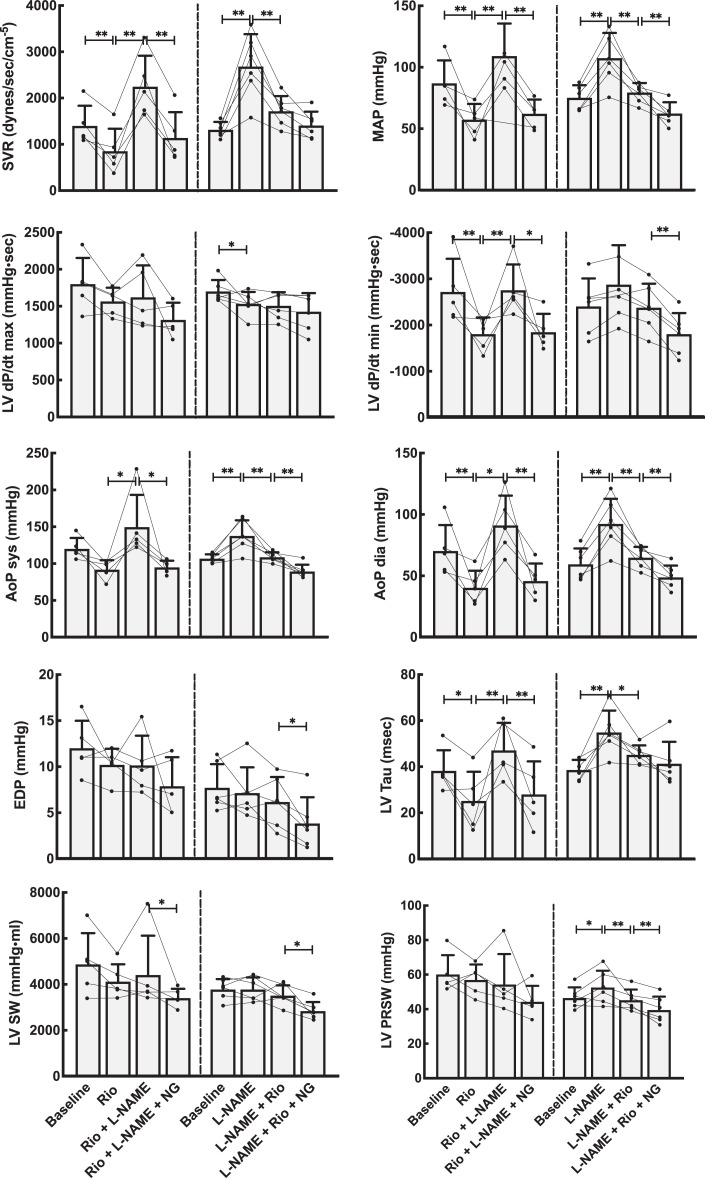
Hemodynamic effects on the systemic circulation by NO modulation using the stimulator riociguat (Rio). Left panels stacked bars show data from the group that received Rio as the first drug, whereas right panels are from the group with primary NO-blockade using l-NAME. Both groups received the NO donor NG as the last drug. Line plots represent the individual animals, while bars are mean values for each combination of medications. Error bars are SD of the mean values at each point. AoPdia indicates diastolic pressure in the aorta; AoPsyst, systolic pressure in the aorta; EDP, end-diastolic pressure; LV dP/dt max and min, maximal left ventricular pressure rises and decline; LV PRSW, preload recruitable stroke work; LV SW, left ventricular stroke work; LV Tau, ventricular relaxation constant; MAP, mean arterial pressure in the aorta; NG, nitroglycerine; SVR, systemic vascular resistance. Linear mixed model statistics. *P < .05, **P < .01.

**Figure 3.**
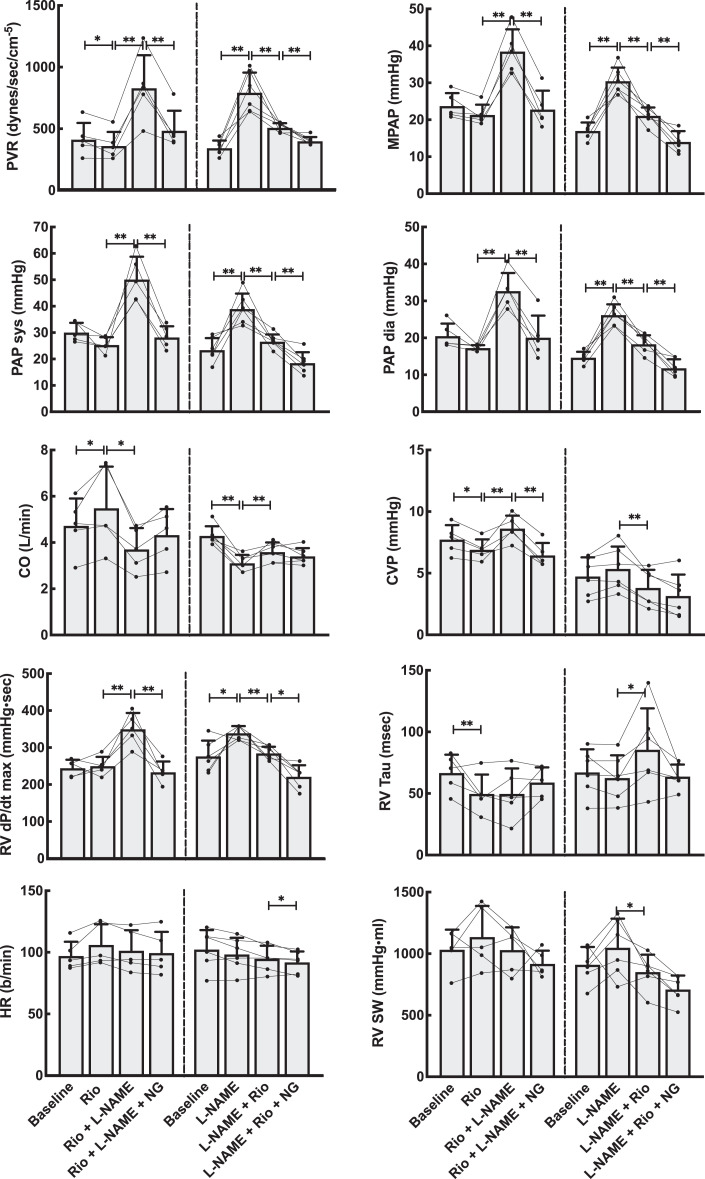
Hemodynamic effects on the pulmonary circulation by NO modulation using the stimulator riociguat (Rio). Left panels show data from the group that received Rio as the first drug, whereas the right panels are from the group where the NO system was first blocked using l-NAME. Both groups received the NO donor NG as the last drug. Line plots represent the individual animals, while bars are mean values for each combination of medications. Error bars are SD of the mean values at each point. CO indicates cardiac output by thermodilution; PVR, pulmonary vascular resistance, PAP sys, PAP dia and MPAP, systolic, diastolic and mean pulmonary artery pressure; RV dP/dt max, maximal right ventricular pressure development; RV Tau, right ventricular relaxation; HR, heart rate; RV SW, right ventricular stroke work. Linear mixed model statistics. *P < .05, **P < .01.

**Figure 4.**
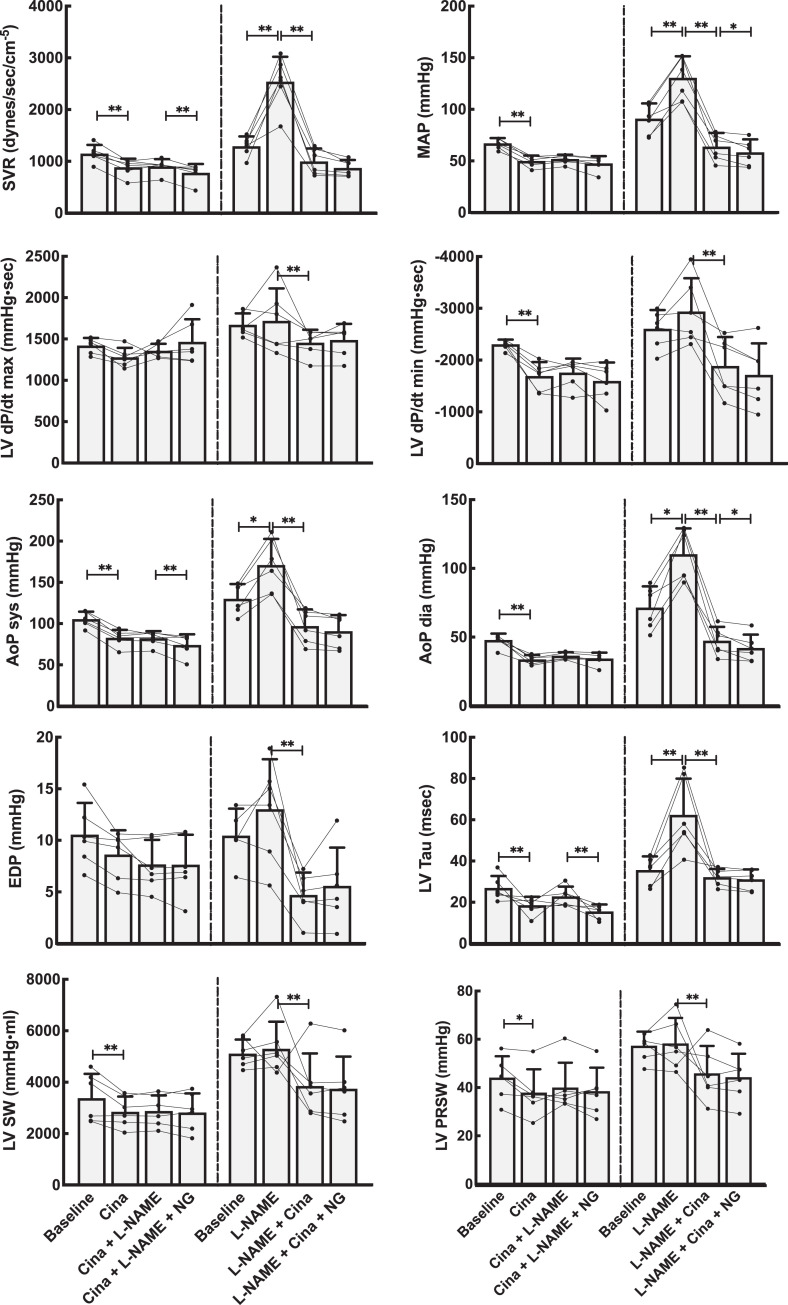
Hemodynamic effects on the systemic circulation by NO modulation using the activator cinaciguat (Cina). Left panels show data from the group that received Cina as the first drug. The right panels show data when the NO system was first blocked using l-NAME. Both groups received the NO donor NG as the last drug. Line plots represent the individual animals, while bars are mean values for each combination of medications. Error bars are SD of the mean values at each point. AoP sys, AoP dia, and MAP, systolic, diastolic, and mean aortic pressure; EDP, end-diastolic pressure; NG, nitroglycerine; LV dP/dt max and min, maximal left ventricular pressure development and reduction; LV PRSW, left ventricular preload recruitable stroke work; LV SW, left ventricular stroke work; LV Tau, ventricular relaxation constant; SVR, systemic vascular resistance. Linear mixed model statistics. *P < .05, **P < .01.

**Figure 5.**
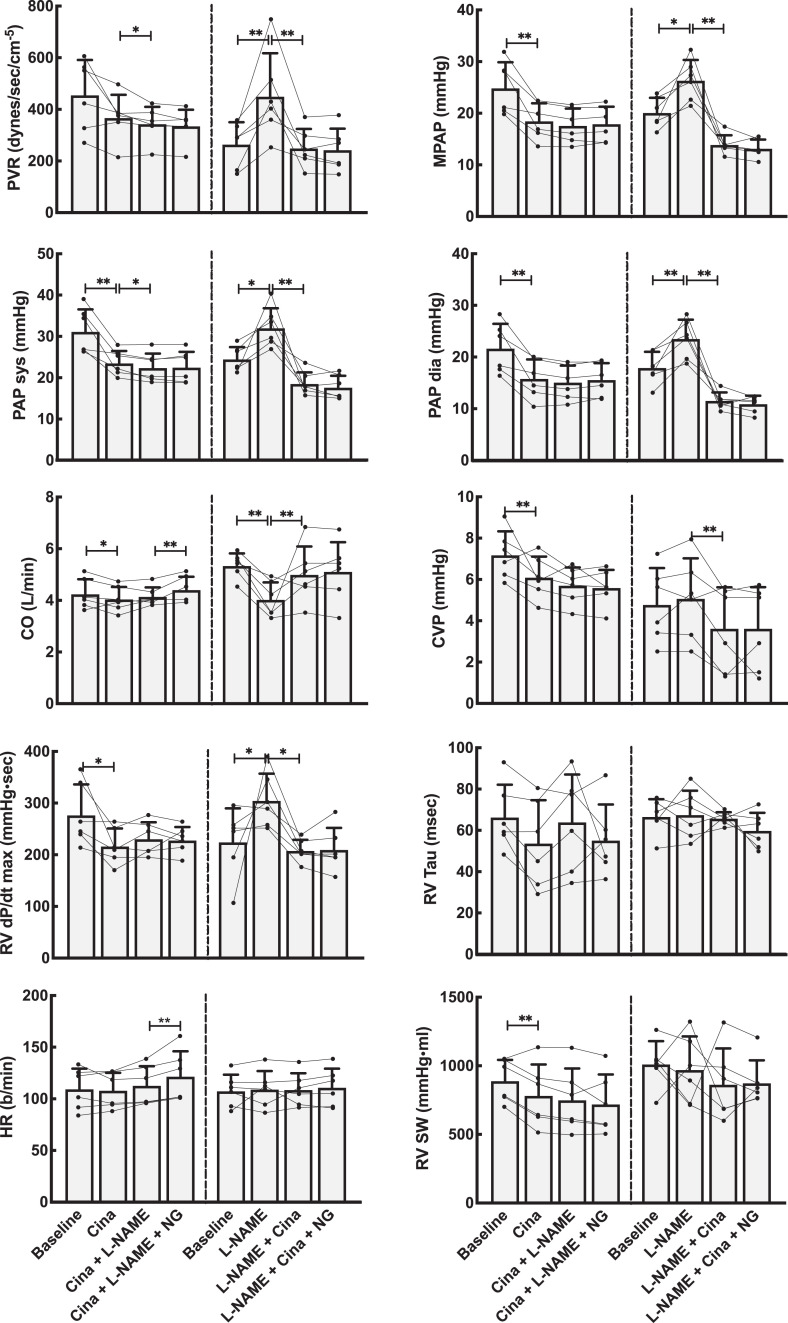
Hemodynamic effects on the pulmonary circulation by NO modulation using the activator cinaciguat (Cina). Left panels show data from the group that received Cina as the first drug, whereas the right panels are from the group where the NO system was first blocked using l-NAME. Both groups received the NO donor NG as the last drug. Line plots represent the individual animals, while bars are mean values for each combination of medications. Error bars are SD of the mean values at each point. CO indicates cardiac output by thermodilution; HR, heart rate; NG, nitroglycerine; PAP sys, PAP dia, and MPAP, systolic, diastolic, and mean pulmonary artery pressure; PVR, pulmonary vascular resistance; RV dP/dt max, maximal right ventricular pressure development; RV SW, right ventricular stroke work; RV Tau, right ventricular relaxation. Linear mixed model statistics. *P < .05, **P < .01.

Basic hemodynamic data for all experimental animals are shown in Figures 2 and 3 (riociguat experiments) and Figures 4 and 5 (cinaciguat experiments). Also shown in these figures are the data after infusion of l-NAME and NG. The interactive effects of riociguat or cinaciguat with NO-modulation are summarized schematically in Table 2.

**Table 2.** Semi-Quantitative Interactive Effects of Riociguat and Cinaciguat Using NO-Modulation.

	Test drug alone		l-NAME alone, Rio-group	l-NAME alone, Cina-group	Test drug after l-NAME		l-NAME after test drug		Nitroglycerin after test drug and l-NAME		Nitroglycerin after l-NAME and test drug	
** **	Rio	Cina	l-NAME	l-NAME	l-NAME + Rio	l-NAME + Cina	Rio + l-NAME	Cina + l-NAME	Rio + l-NAME+ NG	Cina + l-NAME + NG	l-NAME + Rio + NG	l-NAME + Cina + NG
CO	+	−	−	−	+	+	−	NS	NS	+	NS	NS
LVEDP	NS	NS	NS	NS	NS	−−−	NS	NS	NS	NS	−−−	NS
MAP	−	−	++	++	−	−	+++	NS	−−−	NS	−	−
SVR	−	−	++++	+++	−	−−−	+++	NS	−−−	−	NS	NS
LVEF	+	NS	−	−	++	++	−	NS	+	NS	NS	NS
LVSW	NS	−	NS	NS	NS	−	NS	NS	−	NS	−	NS
LV dP/dt max	NS	−	−	NS	NS	−	NS	NS	NS	NS	NS	NS
LV dP/dt min	++	++	NS	NS	NS	++	−−−	NS	++	NS	++	NS
LV Tau	−	−	++	+++	−	−−−	+++	+	−−−	−	NS	NS
LVPRSW	NS	−	+	NS	−	−	NS	NS	NS	NS	−	NS
RAP	−	−	NS	NS	−	−	+	NS	NS	NS	NS	NS
MPAP	NS	−	+++	++	−	−	+++	NS	−−−	NS	−	NS
PVR	−	NS	++++	+++	−	−−−	+++	−	−−−	NS	−	NS
RV dP/dt max	NS	−	+	++	−	−	++	NS	−	NS	−	NS
RV dP/dt min	NS	+	−−−	−−−	++	++	−−−	NS	+++	NS	++	NS
RV Tau	−	NS	NS	NS	++	NS	NS	NS	NS	NS	NS	NS

Abbreviations: AoPdia, diastolic pressure in the aorta; AoPsyst, systolic pressure in the aorta; CO, cardiac output; Cina, cinaciguat; Es, the end-systolic pressure-volume relationship; LV dP/dt max, maximum rate of developed left ventricular pressure during systole; LV dP/min, maximum rate of pressure decay in the left ventricle during diastole; LVEDV, end-diastolic volume in the left ventricle; LVEF, left ventricular ejection fraction; LVESV, end-systolic volume in the left ventricle; LVP developed pressure, pressure difference between maximum and end-diastolic pressure in the left ventricle; LVPed, end-diastolic pressure left ventricle; LVPmax, maximum intraventricular pressure in the left ventricle; LVPRSW, preload recruitable stroke work in the left ventricle; LVSW, left ventricular stroke work; MAP, mean arterial pressure in the aorta; MPAP, mean pulmonary arterial pressure, NG, nitroglycerine; PAPdia, diastolic pressure in the pulmonary artery; PAPsys, systolic pressure in the pulmonary artery; PVR, pulmonary vascular resistance; RAPmean, mean right atrial pressure; Rio, riociguat; RV dP/dt max, maximum rise in right ventricular pressure during systole; RV dP/dt min, maximum pressure decay in the right ventricle during diastole; RVPmax, maximum pressure in the right ventricle; RVPmin, minimum pressure in the right ventricle; RVP developed pressure, the difference between RVPmax and RVPmin; SVR, system vascular resistance. +/− ≤ 25% change, ++/−− = 25% to 50% change, +++/−−− = >51% to 100% change, ++++/−−−− > 100% change from previous combination of medications

### Interactive Effects of Riociguat and NO on Vascular Tone

Compared to vehicle, riociguat reduced the systemic vascular resistance by 40% and induced lower systemic blood pressures. The same dose, with an evident systemic effect, reduced pulmonary vascular resistance by 20% but did not decrease pulmonary systolic or mean pulmonary blood pressures. Cardiac output was slightly increased.

l-NAME resulted in reduced CO, higher systemic and pulmonary blood pressures, and a corresponding calculated increase in systemic vascular resistance (SVR) and pulmonary vascular resistance (PVR), all compatible with its known NO blocking effect. Riociguat, given after l-NAME, increased CO, reduced MAP, SVR, mean pulmonary arterial pressure (MPAP), and PVR demonstrating an NO-independent vasodilatory effect. l-NAME administered after riociguat in separate experiments decreased CO and increased MAP, SVR, MPAP, and PVR, an indication that riociguat interacts with NO in an additive manner.

Finally, nitroglycerine, given after riociguat and l-NAME, resulted in unchanged CO, decreased MAP, SVR, systolic and diastolic pulmonary pressures, MPAP, and PVR confirming that NO retains its dose-dependent effect also after giving riociguat.

### Interactive Effects of Cinaciguat and NO on Vascular Tone

Introduced in untreated animals, cinaciguat lowered systemic and pulmonary systolic, diastolic, mean, and venous blood pressures. Cardiac output was slightly reduced. Also, calculated SVR decreased, while PVR remained unchanged. Thus, cinaciguat, as riociguat, shows no selective pulmonary vascular dilatory effect.

Cinaciguat given after the NO-blocker l-NAME induced both systemic and pulmonary vasodilation. However, l-NAME, given after cinaciguat, had almost no vascular effect, indicating that the effect of cinaciguat could not be modulated by altering NO-tone. This was confirmed by giving nitroglycerine directly after cinaciguat, as this had almost no vasoactive effect, as shown in Table 2 and Figures 4 and 5.

### The Effects of Riociguat and Cinaciguat on Cardiac Function

The effects of riociguat and cinaciguat on integrated cardiac function were tested in load-dependent and independent calculations summarized in Figure 2 and 4 and Table 2. The load-related parameters of time-dependent pressure development were slightly reduced for both left and right ventricle. However, calculating the load-independent parameters of preload recruitable stroke work showed that riociguat in this dose had a neutral effect on cardiac contractility. Cinaciguat induced a small reduction also on the load-independent PRSW in the left ventricle.

Both riociguat and cinaciguat demonstrated a slower maximum intraventricular pressure fall rate, expressed as dP/dt min in the left ventricle. For the right ventricle, cinaciguat gave a small decrease in the absolute value of dP/dt min, while riociguat did not have any effect on this index. Using the Tau-index to curve fit the pressure decay during diastole, both cinaciguat and riociguat resulted in significantly faster relaxation of the left ventricle compared to baseline, and both cinaciguat and riociguat counteracted the effect on isovolumic relaxation seen after l-NAME by again reducing the Tau index. Both cinaciguat and riociguat demonstrated unchanged LV end-diastolic volumes at lower end-diastolic pressures.

## Discussion

We have shown that both cinaciguat and riociguat have pronounced vasodilatory properties in the normal systemic vasculature. However, in these healthy juvenile pigs, these compounds have only minor direct effects in the pulmonary circulation. After NO-blockade with l-NAME, a vasodilatory effect in both vasculatures was unmasked. Importantly, no vasoactive effect of NO could be observed after cinaciguat infusion, indicating a functional NO-blocking effect of this compound. In contrast, NO modulation after riociguat infusion altered vascular tone in an interactive, qualitative physiological expected manner. The direct cardiac responses of both drugs were dominated by their unloading effects, and only a small possible reduction in load-independent contractility observed from cinaciguat. The small lusitropic effect for both drugs may also be related to the unloading effect from their vasodilation.

The interplay of riociguat and cinaciguat with the NO-system in the pulmonary circulation has previously been studied primarily in pulmonary arterial hypertension (PAH) models.^21-26^ These observations are somewhat in contrast with the relative weak pulmonary effects in healthy animals observed in our study. A possible explanation might be a different level of the sGC redox-forms in pathological and normal pulmonary vessels. Soluble guanylate cyclase is upregulated in human idiopathic pulmonary hypertension and animal models of PAH,^25^ and the vasodilatory effect of both sGC stimulators and activators is augmented by the oxidation of sGC. It is not known what the proportion of the different oxidation levels of sGC is in intact, healthy animals. The effects of these drugs given intravenously in intact animals and the interactions with NO are consequently unknown. Our study using cinaciguat indicates that there is a functionally oxidized sGC also in the healthy systemic circulation, but the activity seems to be low in the pulmonary vasculature. An alternative explanation for the small effects of these drugs in the pulmonary vasculature is the probable normal level of nitric oxide synthase and a concomitant higher activity in healthy animals compared to reduced levels in pulmonary vascular pathology.^27^

In a clinical study with riociguat applied in pulmonary hypertension secondary to diastolic heart failure, but without increased PVR, there was no change in transpulmonary pressure gradient or pulmonary vascular resistance.^28^ The explanation for this might be a lower expression of and lower oxidation levels of sGC in the lungs with no primary pathology, abolishing any pulmonary vascular selectivity when the drug is applied intravenously. Inhalation, in contrast to intravenous administration of sGC-activators and stimulators, has been shown to give selective pulmonary vasodilatation in awake lambs with acutely thromboxane-induced pulmonary hypertension.^26^ Still, such an application has not to our knowledge been tested in humans or healthy animals.

The dynamics between NO, sGC activators, sGC stimulators, and sGC have been studied in cell cultures with outcomes indicating that sGC activators can skew the redox balance between a small pool of oxidized sGC in the direction of increased oxidation, rendering the sGC insensitive to NO.^29^ An in vitro study on healthy porcine coronary arteries and rat thoracic aortas have shown that cinaciguat has a profound vasodilatory effect even in the absence of vascular pathology.^30^ In that study, cinaciguat exhibited an irreversible effect, and the authors proposed that cinaciguat shift the red-ox-equilibrium of sGC toward the heme-free species. This, in turn, could explain the observed insensitivity to NO-manipulation after cinaciguat in our study. The time span for potential recovery of this effect is unknown.

A phase II study on cinaciguat in acutely decompensated heart failure was stopped prematurely due to the observation that 71% of patients experiencing adverse events, mostly hypotension. The prolonged action and loss of NO-system modulation seem to make cinaciguat a potent but difficult drug to master in clinical practice. There are no ongoing patient studies with cinaciguat,^31^ but there are 9 actively recruiting studies on riociguat in scleroderma, sickle cell disease, PAH, and chronic thromboembolic pulmonary hypertension.^32^ As seen from our research, the sGC activators have profound and partly unphysiological effects demanding considerable preclinical assessments before clinical application. There are some phase 1 studies ongoing with sGC activators in patients with chronic kidney failure, PAH, and acquired pulmonary distress syndrome.^33^ These studies need to be monitored closely for possible adverse circulatory effects.

We did not observe any effect of riociguat on cardiac contractility evaluated by preload recruitable stroke work and only a small negative effect of cinaciguat. Studies on ischemia–reperfusion injuries in the heart have shown cytoprotective properties of both cinaciguat and riociguat but no fundamental change in heart function.^11,34-36^ Such a protective effect can potentially be related to myocardial calcium handling.^37,38^ Infusion of NO donors to the coronary circulation seems to work in a biphasic and concentration-dependent fashion with increased contractility and chronotropy in low normophysiological concentrations and the opposite effect at higher concentrations.^39^ Therefore, the effect of exogenous NO through NO donors might be hard to predict, and since the direct activation or stimulation of sGC additionally bypasses the cGMP-independent effects of NO, they will predictably have a different pharmacological profile compared to endogenous NO stimulation.^39^

In our study, the most pronounced diastolic effect was a shortened LV relaxation, expressed in the Tau index. The maximum pressure fall rate in the left ventricle also decreased concomitantly with the Tau index reduction indicating an unloading effect of both riociguat and cinaciguat.^40^ In a recent paper using the sGC stimulator Bay 41-8543 in an experimental rat model of HEFPEF (Heart Failure with Preserved Ejection Fraction), Wilck and coworkers observed a normalization of diastolic dysfunction.^41^ Of particular clinical interest, the phase 3 study of Vericiguat in HEFPEF still awaits conclusion.^42^

## Methodological Aspects

The pigs were sedated throughout the experiments with no concomitant use of muscle relaxant. Midazolam has a negative inotrope and chronotrope effect in high but not in clinically relevant anesthetic doses.^43^ Pentobarbital have negative chronotropic and inotropic effects in anesthetic doses and results in reduced blood pressure, stroke volume, and central venous pressure.^44^ Furthermore, pentothal has direct effects on vascular smooth vasculature.^45^ The addition of Fentanyl induces no major hemodynamic effects but attenuate pain-mediated stress responses.^46^ All these effects influence critical target values in hemodynamic experiments. The goal of the anesthesia protocol was to create a stable hemodynamic environment for the course of the experiment to be able to compare the different stages of the experiment against each other. This protocol has proven to give good stability in prolonged hemodynamic experiments in our lab.^47^ Along the same line of reasoning, hexamethonium attenuates sympathetic activation during interventions and isolates the drug effects at each stage of the experiment. As a trade-off, however, hexamethonium blocks a normal heart-rate response to hypotension.

The dose target for both riociguat and cinaciguat was chosen to keep MAP above 50 mm Hg. We chose a high dose with clear hemodynamic effects in order to induce clear discernable effects of these drugs without the need for excessive amounts of animals to be used in the protocols. In the main study, MAP after cinaciguat infusion was 50 ± 5 mm Hg and in the riociguat group 57 ± 13 (*P* = 0.25). There was no significant difference in SVR, PVR, or MPAP after giving riociguat or cinaciguat. The perfusion pressure in the cinaciguat group is closer to the limit of autoregulation of coronary perfusion pressure^48^ than in the riociguat group and may have attenuated cardiac performance predominantly in the cinaciguat group.

We used PRSW as a contractility index. End-systolic pressure–volume relationship and maximum dP/dt are alternative indexes, but PRSW have been shown to be more load-independent and more reproducible than these alternative indexes.^49,50^

## Conclusion

In summary, both cinaciguat and riociguat have vasodilatory properties in the healthy systemic circulation but only a weak effect in the pulmonary vasculature. After blocking NO production with l-NAME, both drugs demonstrate vasodilatory effects in both vasculatures. Furthermore, after giving cinaciguat NO modulation is without any effect on vascular resistance, indicating a functional block of the active NO sites on all forms of sGC. The pharmacological profile of this sGC-activator is, therefore, indiscriminative and, as such, is a difficult drug to handle in a clinical setting. Soluble GC activators seem to have a long way to potential clinical applications.
